# Innovations in Biofilm Prevention and Eradication in Medical Sector: An Integrative Review

**DOI:** 10.3390/pathogens14121242

**Published:** 2025-12-04

**Authors:** Konrad Niedźwiadek, Magdalena Polak-Berecka, Adam Waśko

**Affiliations:** 1 Department of Biotechnology, Microbiology and Human Nutrition, Faculty of Food Science and Biotechnology, University of Life Sciences in Lublin, Skromna 8, 20-704 Lublin, Poland; magdalena.polak-berecka@up.lublin.pl (M.P.-B.); adam.wasko@up.lublin.pl (A.W.); 2 Research and Development Department, Margomed S.A., 16 Erazma Plewińskiego Street, 20-270 Lublin, Poland

**Keywords:** antimicrobial surfaces, bacteriophage-based strategies, biofilm eradication, enzymatic coatings, medical device infections, nanotechnology, photodynamic therapy

## Abstract

Background: Biofilm-associated infections remain a major challenge in modern medicine due to their high resistance to antibiotics and immune defences. Advances in materials science, chemistry, and nanotechnology have led to the development of innovative, non-antibiotic approaches to prevent or eradicate biofilms. Methods: This review summarises antibiofilm strategies reported between 2020 and 2025, grouped into chemical, enzymatic, physical–photonic, nanomaterial-based, and biological hybrid categories. Results: Chemical methods such as silver-based chemical systems, nitric oxide donors, and biosurfactants disrupt bacterial membranes, generate reactive oxygen species, and inhibit quorum sensing. Enzymatic coatings with DNase I or lysostaphin effectively reduce *Staphylococcus aureus* and *S. epidermidis* biofilms, showing stability after sterilisation and high biocompatibility. Physical–photonic techniques, including photocatalytic and light-activated coatings, provide controllable and renewable antibacterial activity. Nanomaterials such as silver nanomaterials, chitosan-based carriers, magnetic ferrites, and catalytic nanozymes enable targeted, ROS-mediated biofilm disruption. Biologically derived systems, including bacteriophage hydrogels and plant metabolites, offer eco-friendly, biocompatible alternatives. Conclusions: Recent antibiofilm innovations mark a transition from conventional antibiotics to multifunctional and adaptive systems integrating chemical, enzymatic, and physical mechanisms for effective biofilm control on medical surfaces.

## 1. Introduction

Biofilms are structured microbial communities enclosed in self-produced polymeric matrices that adhere to biotic and abiotic surfaces. Within these matrices, bacteria exhibit up to thousand times higher tolerance to antibiotics than planktonic cells, posing a major challenge in clinical contexts such as catheters, implants, ventilator circuits, and wound dressings. Bacteria within a biofilm communicate through quorum sensing, a coordinated signalling strategy involving the production, release, and detection of small diffusible molecules that enable the collective regulation of population density dependent behaviours [1]. Conventional antimicrobial therapies often fail because extracellular polymeric substances (EPS) act as diffusion barriers, limit antibiotic penetration, and provide a protective environment that favours horizontal gene transfer and persistence [2]. Moreover, biofilm formation promotes the emergence of multidrug-resistant (MDR) phenotypes, contributing to nosocomial infections with *Staphylococcus aureus*, *Pseudomonas aeruginosa*, *Klebsiella pneumoniae*, and *Escherichia coli* [3].

Given this complexity, current research has shifted from attempting to eradicate biofilms solely with antibiotics to developing non-antibiotic, surface-based, and multifunctional strategies. These include (i) chemical systems (e.g., nitric oxide donors, antimicrobial peptides, biosurfactants, and natural extracts) that alter bacterial adhesion and physiology; (ii) enzymatic systems that degrade structural components such as DNA or peptidoglycan; (iii) physical and photonic systems that harness light, heat, or oxidative fields to deactivate microbes; and (iv) nanomaterial or hybrid biological systems that combine several mechanisms in a single platform [4,5,6]. While previous reviews have predominantly addressed isolated anti-biofilm strategies, such as quorum-sensing inhibition, antimicrobial peptides, or surface modification, there has been a lack of integrative analyses linking chemical, biological, and physical innovations across disciplines. This work fills this gap by synthesising recent advances into a unified framework focused on their applicability in medical device technology.

This review aimed to address the research question concerning which innovative strategies and technologies are currently being implemented in medical settings to prevent and eradicate biofilms. The following work considers a multidisciplinary approach, including chemical, biological, and physical solutions and their combinations. Particular attention was paid to solutions with real potential for implementation in the medical sector. In this review, we have integrated the results from recent studies, summarising their mechanisms, experimental outcomes, and perspectives, while highlighting the most promising technologies for preventing device-associated infections.

## 2. Materials and Methods

### 2.1. Protocol

To ensure methodological transparency, minimise selection bias, and facilitate a rigorous synthesis of scientific data, the review was planned in accordance with the Preferred Reporting for Systematic Reviews and Meta-Analyses (PRISMA 2020) [7] and the integrative review method developed by Whittemore and Knafl (2005) [8]. The free Rayyan (Rayyan Systems Inc., Doha, Qatar) tool was used to facilitate the screening and selection of publications.

To explore the thematic structure of the included literature, a bibliometric keyword co-occurrence analysis was performed using VOSviewer version 1.6.20 (Centre for Science and Technology Studies, Leiden University, Leiden, The Netherlands). The analysis was conducted exclusively on the final set of eligible studies and applied full counting with a minimum keyword occurrence threshold of two, selected to accommodate the size of the dataset. The resulting concept map enabled the visualisation of co-occurrence patterns across author keywords and facilitated the identification of bibliometrically defined research clusters related to innovations in biofilm prevention and eradication.

### 2.2. Information Sources and Search Strategy

A comprehensive literature search was conducted in four major databases: PubMed (https://pubmed.ncbi.nlm.nih.gov, accessed on 29 June 2025), Scopus (https://www.scopus.com, accessed on 29 June 2025), and Web of Science (https://www.webofscience.com, accessed on 29 June 2025). The search was limited to publications from 2020 to 2025 to focus on recent advances and increase the likelihood of identifying innovative approaches. The following combinations of keywords were used: (biofilm OR biofilm formation OR biofilm eradication) AND (innovation OR novel therapy OR nanotechnology OR photodynamic OR enzyme OR bacteriophage OR coating OR quorum sensing) AND (medical device OR clinical infection OR wound OR catheter OR implant) AND NOT (review). To ensure accurate and objective study selection, search results from each database were exported in the RIS format and imported into the Rayyan platform for independent screening.

### 2.3. Eligibility Criteria

Studies evaluating the efficacy or investigating the mechanism of action of novel approaches for biofilm prevention and elimination in the medical field were considered eligible for screening. To advance to the subsequent stages of the review process, the publications were required to address clinical or experimental applications, particularly in the context of medical devices. Both in vitro studies and those conducted on animal models were included, provided they were relevant to medical applications. The detailed inclusion and exclusion criteria are summarised in Table 1.

### 2.4. Selection Process

All records retrieved from the database search were exported in .bib or .txt format and imported into the Rayyan platform. Rayyan automatically detected and flagged duplicate entries, which were subsequently reviewed and removed before the screening phase. A total of 156 duplicates were excluded at this stage, ensuring that each study was assessed only once.

The selection process was conducted in two subsequent stages. In the first stage, titles and abstracts were screened to assess their relevance based on the predefined inclusion criteria. Publications that met the initial criteria were then subjected to full-text evaluation to verify compliance with both content-related and methodological requirements. Screening and selection were independently performed by the three authors using Rayyan, and any discrepancies were resolved through discussion and consensus.

### 2.5. Data Synthesis

A formal meta-analysis was not performed because of the significant methodological and thematic heterogeneity of the included studies. Instead, a narrative synthesis approach was adopted, allowing for qualitative analysis and organisation of findings within predefined thematic categories. Publications were grouped according to the main types of antibiofilm strategies, which included material-based approaches (such as antibacterial coatings and nanoparticles), biological agents (including bacteriophages and enzymes), chemical agents, physical methods, and holistic strategies, involving combinations of substances or integration of chemical/biological agents with physical methods.

To facilitate the structured analysis and comparison of diverse strategies for biofilm prevention and eradication in medical contexts, a comparative matrix was developed as a tool for synthesising data across studies with different methodologies and focus areas. This matrix accounted for key parameters, such as the type of innovation and its mechanism of action, application context, reported efficacy, and the main advantages and limitations identified by the authors.

In addition to extracting information reported in the primary studies, the authors implemented an independent integrative framework to harmonise and interpret heterogeneous evidence. First, a unified classification scheme for antibiofilm innovations was developed, consolidating diverse technologies into categories. Second, efficacy outcomes, reported using non-comparable methodological metrics, were standardised into qualitative tiers to enable cross-study comparison. Third, advantages and limitations were synthesised using an author-defined coding system that grouped study-specific observations into broader evaluative themes. Finally, the authors introduced an additional dimension of analysis focused on applicability, assessing feasibility, safety considerations, and technological aspects on the basis of expert judgement. Collectively, Table 2 reflects not only published findings but also the authors’ structured analytical contribution, providing a coherent and comparative synthesis across heterogeneous antibiofilm strategies.

## 3. Results and Discussion

### 3.1. Summary of Studies

**Figure 1:**
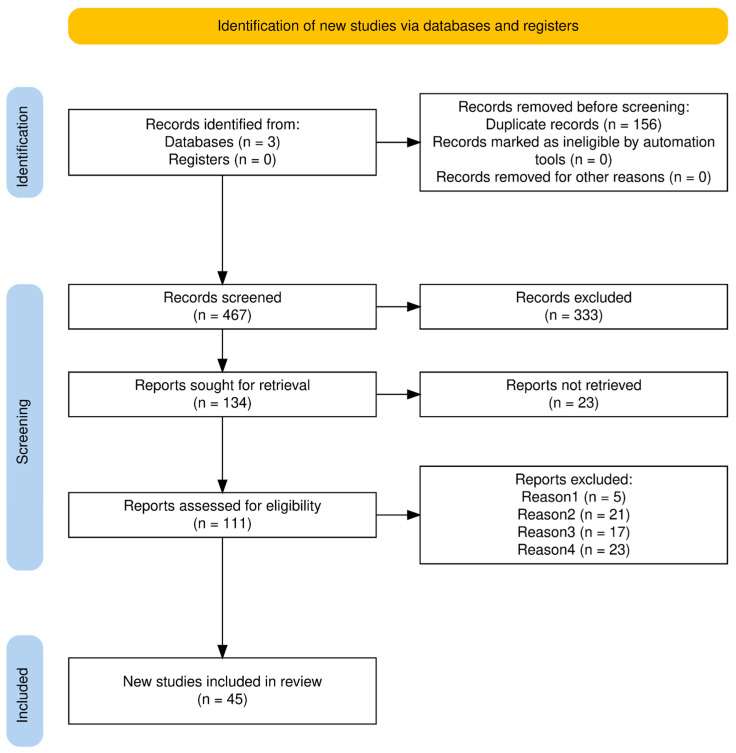
PRISMA flow diagram summarising the article screening process and reasons for exclusion. Reason 1—Not published in English, Reason 2—Full text not available, Reason 3—Focuses on biofilms outside the medical sector (e.g., food industry); Reason 4—Does not describe an innovative approach.

The study selection process, conducted in accordance with the PRISMA 2020 guidelines, identified a total of 623 records across three databases: PubMed (*n* = 260), Web of Science (*n* = 98), and Scopus (*n* = 265) (Figure 1). Following the removal of 156 duplicates, 467 publications were deemed eligible for the initial screening stage, of which 333 were excluded based on an analysis of the titles and abstracts. Consequently, 134 reports were selected for full-text evaluation; however, 23 were inaccessible, leaving 111 to be assessed for compliance with the inclusion criteria. At this stage, 66 papers were excluded for the reasons shown in Figure 1 (Reason 1: *n* = 5; Reason 2: *n* = 21; Reason 3: *n* = 17; and Reason 4: *n* = 23). Ultimately, 45 studies that met the eligibility criteria were included in this review.

**Figure 2:**
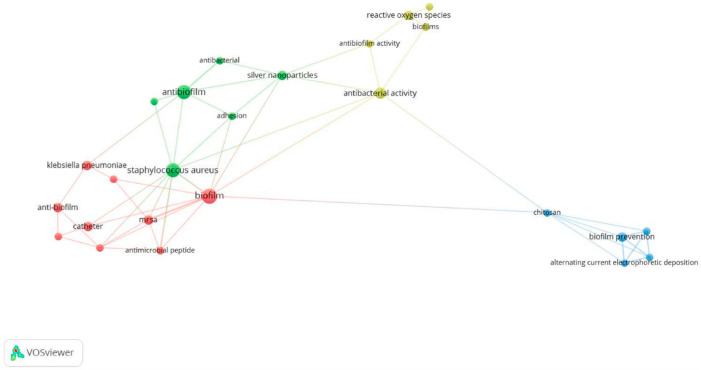
Keyword co-occurrence network generated using VOSviewer version 1.6.20 (full counting; minimum occurrence = 2; normalisation: association strength). The visualization reveals four thematic clusters: the red cluster, representing clinically relevant pathogens and device-associated biofilm formation; the green cluster, reflecting microbial adhesion and nanomaterial-based antimicrobial strategies; the yellow cluster, corresponding to oxidative and reactive oxygen species–mediated antibiofilm mechanisms; and the blue cluster, denoting polymer-based and surface-engineering approaches for biofilm prevention.

VOSviewer was used to generate a keyword co-occurrence map, which allowed for the graphical visualisation of the dominant thematic areas and their relationships (Figure 2).

The bibliometric visualisation revealed four thematic clusters characterising recent antibiofilm research in the medical context. The red cluster (including anti-biofilm, catheter-related terms, *Klebsiella pneumoniae*, MRSA, and antimicrobial peptides) represents studies focused on clinically relevant pathogens and device-associated biofilm formation. The green cluster links *Staphylococcus aureus*, adhesion, antibiofilm and antibacterial activity, and silver nanoparticles, reflecting literature addressing microbial adhesion and nanomaterial-based antimicrobial approaches. The yellow cluster, centred on antibacterial and antibiofilm activity together with reactive oxygen species, corresponds to research exploring oxidative and ROS-mediated mechanisms of biofilm disruption. The blue cluster (dominated by chitosan, biofilm prevention, and electrophoretic deposition techniques) denotes polymer-based and surface-engineering approaches aimed at preventing biofilm formation. Overall, the network illustrates the diversity of research themes within the included studies and indicates moderate bibliometric connections among them, without implying mechanistic or hierarchical relationships between the identified technological approaches.

In recent years, significant progress has been made in the development of innovative strategies for the prevention and eradication of medical biofilms, which can be broadly classified into chemical, physical, and biological approaches. To facilitate the structured analysis and comparison of diverse strategies for biofilm prevention and eradication in medical contexts, a comparative matrix was developed. This matrix was used to synthesise data across studies (Table 2). The interpretation of the evidence in Table 2 applies an author-defined analytical framework that standardises heterogeneous outcomes and organises technologies into coherent categories, thereby revealing mechanisms of action and applicative relevance absent from the primary studies.

A detailed discussion of various innovative technologies in terms of their potential and limitations in combating biofilms in medical environments is presented below.

### 3.2. Chemical Strategies and Nanomaterials-Based Systems

#### 3.2.1. Silver-Based Systems

The most established example of a chemical antibiofilm design combines antiadhesive and contact-killing mechanisms using silver nanoparticles (AgNPs). In a urinary catheter model infected with multidrug-resistant (MDR) *E. coli* U12, AgNPs disrupted bacterial membranes, caused cytoplasmic leakage, and inhibited EPS synthesis via reactive oxygen species (ROS) activity, with a minimum inhibitory concentration (MIC) of approximately 85 µg/mL [4]. Crystal violet (CV) staining and scanning/transmission electron microscopy analyses confirmed a strong reduction in biofilm biomass and surface adhesion. The coating demonstrated polymer compatibility, suggesting its feasibility for urinary devices; however, translation requires precise control of Ag^+^ release, full ISO 10993-1 [48] biocompatibility assessment, and validation of coating durability under synthetic urine flow and ethylene oxide (EO) sterilisation. Selem et al. [4] further proposed coupling AgNPs with light-responsive systems such as photodynamic therapy to enable on-demand activation.

Hybrid photocatalytic coatings of silver/titanium oxides (Ag/TiO_x_) extend this approach by integrating anti-adhesive properties with light-induced reactive oxygen species (ROS) generation [4]. These coatings achieved >80% biomass reduction for *S. aureus* and *E. coli* under UV or visible light, while minimising Ag^+^ leaching (<0.1 ppm) and cytotoxicity [3]. The synergy of photocatalytic TiO_2_ and silver provides dual antibacterial activity, ROS-mediated oxidative killing, and ionic disruption, offering sustained antibiofilm performance over multiple illumination cycles. The main challenges are ensuring sufficient light penetration in clinical settings and preventing substrate photodegradation.

Hydrophilic AgNP-hybrid coatings add an anti-adhesive hydrophilic matrix to silver’s contact-killing. Peter et al. [13] demonstrated >70–80% reduction in cell attachment and >80% biomass reduction on polymeric surfaces exposed to *E. coli*, *S. aureus*, and *S. epidermidis*. The coatings retained their hydrophilicity after multiple washing cycles, showed negligible Ag^+^ release, and exhibited mechanical durability suitable for reusable devices. However, the gradual loss of wettability and microdamage under long-term mechanical stress remain limiting factors.

#### 3.2.2. Antimicrobial Peptides and Nitric–Oxide Hybrids

Cationic antimicrobial peptides (AMPs) are leading molecular candidates for antibiotic-free infection control. The hybrid peptide CM-10K14K prevented *S. aureus*, *S. epidermidis*, and *E. coli* biofilms on Foley catheter materials with low cytotoxicity, maintaining activity after washing cycles [19]. Similarly, RK22, a synthetic broad-spectrum AMP, induced membrane depolarisation and QS inhibition, resulting in 85–95% biomass reduction and sustained efficacy for at least 48 h, with MIC ≈ 6.25 µg/mL for *S. aureus* and minimal fibroblast toxicity [25].

A multifunctional approach was achieved using FOTyr-AMP, an AMP conjugated with a nitric oxide (NO) donor. By combining mechanical membrane disruption with NO-mediated EPS dispersion, this compound eliminated >90% of *S. aureus* and *E. coli* biofilm biomass in vitro and achieved complete removal with reduced inflammation in vivo [23]. The synergy between cationic peptides and controlled NO release exemplifies the transition from simple antimicrobials to smart antibiofilm molecules that can be therapeutically modulated.

#### 3.2.3. Cationic Polymer Coatings

Permanent cationic coatings, such as poly[2-(dimethylamino)ethyl methacrylate] (DMPEI), kill bacteria via electrostatic membrane disruption without leaching of the coating. On PVC catheters, DMPEI reduced the *S. aureus*, *E. coli*, and *Candida albicans* biofilm biomass by 80–95% [30]. SEM and CLSM imaging confirmed the structural collapse of biofilms, while ISO 10993-5 [49] cytotoxicity tests showed fibroblast compatibility at ≤0.1 mg/mL. Contact-active polymers, such as DMPEI, offer durable antibacterial surfaces; however, precise control of the coating thickness and mechanical impact is necessary.

#### 3.2.4. Curcumin–Chitosan Magnetic Nanocarriers

Nanocomposite systems integrating natural polymers with active agents represent a versatile platform for biofilm prevention and eradication. Curcumin–chitosan nanocarriers, developed by Salazar-Sesatty et al. [14], exemplify this class of hybrid nanomaterials. These carriers combine the antimicrobial and anti-QS properties of curcumin with the cationic, anti-adhesive, and biocompatible characteristics of chitosan.

Mechanistically, curcumin disrupts bacterial membranes, induces oxidative stress, and suppresses EPS synthesis and QS. Chitosan provides a positively charged surface that interacts electrostatically with bacterial cell walls, inhibiting their adhesion and promoting local retention. The magnetic core of the system allows for external control and targeted localisation on implant surfaces using magnetic fields, thereby increasing local drug retention and site-specific activity.

In in vitro models using MRSA and coagulase-negative staphylococci (CoNS), the formulation significantly reduced biofilm biomass and cell viability, with enhanced effects when combined with oxacillin, demonstrating synergistic antibacterial action [14]. Cytocompatibility assays confirmed the absence of toxicity to mammalian cells, supporting its biomedical applicability.

This “smart” nanocarrier design allows for magnetic guidance, controlled release, and integration with implant coatings. However, scalability challenges remain, particularly in the standardisation of release kinetics, sterilisation compatibility (EO or γ-irradiation), and stability during storage. Despite these challenges, curcumin–chitosan nanocarriers provide a promising blueprint for multifunctional antibiofilm delivery systems that merge natural actives, responsive control, and implant compatibility into a single platform.

#### 3.2.5. Ni–Cu–Zn Ferrite Nanomaterials

Inorganic Ni–Cu–Zn ferrite nanostructures offer a non-leaching, long-term antibiofilm mechanism based on contact-active and catalytic effects, rather than biocide release [18]. Designed as magnetic coatings for biomedical surfaces, these ferrites exhibit intrinsic antibacterial properties and allow potential magnetic manipulation.

Their mechanism involves direct membrane damage via catalytic redox cycling, which generates ROS, particularly hydroxyl radicals (•OH) and superoxide anions (O_2_^−^). Transition metal ions (Ni^2+^, Cu^2+^, Zn^2+^) enhance oxidative stress and destabilise bacterial membranes, whereas the surface rigidity and high conductivity of the material reduce bacterial adhesion and limit EPS stabilisation.

In in vitro tests with *E. coli* and *S. aureus*, Ni–Cu–Zn ferrite coatings achieved >90% reduction in CFU counts and biofilm biomass. The activity was retained after multiple exposure cycles, indicating long-term functionality [18]. Importantly, no significant metal-ion leaching was observed, confirming that the antibacterial action is contact- and catalysis-based rather than release-driven.

The key advantages of these systems include their durability and reusability without the need for regeneration, as well as their compatibility with both metallic oxides and polymeric substrates. An additional benefit is their potential for integration into magnetically responsive composites, which enables external-field activation and further expands their functional versatility.

However, the cytotoxicity concerns associated with nickel and copper necessitate thorough ISO 10993-17/-18 [50,51] testing for metal ion migration and biocompatibility. Further assessment of long-term ageing, sterilisation, and corrosion stability is required. Despite these challenges, Ni–Cu–Zn ferrites stand out as a sustainable, antibiotic-free alternative to leaching coatings, offering persistent antibacterial protection and the potential for magnetic-assisted control.

#### 3.2.6. Alginate–Kaolin–Silver Bio-Nanocomposites

The sodium alginate–kaolin–Ag nanoparticle composite developed by Imani et al. [24] integrated natural and inorganic components into a multifunctional, biocompatible matrix. Alginate provides a flexible, biodegradable hydrogel network; kaolin contributes mechanical reinforcement and adsorption capacity; and silver nanoparticles (AgNPs) deliver bactericidal efficacy through reactive oxygen species (ROS) generation and membrane disruption [24].

This tri-component system achieves both barrier and contact-killing effects. The alginate-kaolin framework stabilises the silver nanoparticles and modulates their release kinetics, preventing uncontrolled Ag^+^ diffusion and maintaining long-term antimicrobial performance. Protein adsorption and limited permeability further restrict bacterial adhesion and nutrient transport, thereby amplifying antibiofilm efficiency.

In in vitro assays against *Streptococcus mutans*, *Escherichia coli*, and *Staphylococcus aureus*, the composite achieved >90% reduction in biofilm biomass and significant CFU decreases compared with unmodified controls. SEM and EDS confirmed the uniform dispersion of AgNPs within the polymer clay matrix [24]. The material exhibited high mechanical stability, elasticity, and excellent cytocompatibility in fibroblast and haemolysis assays.

The advantages of this composite include solvent-free, water-based synthesis, biodegradability, and tunable silver content, allowing optimisation between efficacy and biocompatibility. The main limitations are the standardisation of Ag^+^ release kinetics, evaluation of long-term stability under humid and sterilisation conditions, and verification of compliance with ISO 10993-1 [48]. Alginate–kaolin–Ag composites exemplify how natural–inorganic hybrids can achieve robust antibacterial and antibiofilm performance while meeting biocompatibility and environmental sustainability

#### 3.2.7. Alginate–Polymer Nanozymes and Catalytic Nanostructures

In addition to passive composites, certain nanomaterials exhibit enzyme-like catalytic functions (nanozymes). Liang et al. [6] designed Carbon Dots@PtNPs (CPP nanozymes) that mimic peroxidase activity, converting hydrogen peroxide (H_2_O_2_) into hydroxyl radicals (•OH). These ROS oxidise proteins, polysaccharides, and nucleic acids within the biofilm matrix, leading to bacterial death and structural collapse.

In both in vitro and in vivo models involving *Staphylococcus aureus* and *Pseudomonas aeruginosa* biofilms, CPP nanozymes eradicated >99% of biofilm biomass and bacterial cells. In a murine skin infection model, they promoted complete MRSA biofilm removal and accelerated wound healing without observable tissue damage [6]. The nanozymes maintained their catalytic efficiency over a wide pH (5–8) and temperature (25–40 °C) range, confirming their functional stability.

The dual role of CPP nanozymes, as antibacterial and tissue-regenerative agents, makes them highly attractive for wound-healing dressings and implant coatings. Nonetheless, ROS control is critical for preventing excessive oxidative stress and host tissue damage. Long-term biodegradation and pharmacokinetics must also be clarified for clinical use.

Nanozyme systems, such as CPP, highlight the future direction of enzyme-inspired nanomaterials that merge catalytic precision with nanostructural robustness to achieve antibiotic-free biofilm eradication.

Nanomaterial-based systems share several distinguishing traits that make them particularly promising for antimicrobial applications. They exhibit multimodal activity by combining ROS generation, contact killing, and anti-adhesive functions. Their development often emphasises sustainability through the use of natural polymers, such as alginate and chitosan, as well as solvent-free synthesis routes. Moreover, these systems display smart functionalities, including magnetic, photothermal, or catalytic responsiveness, which allows precise external control of their activity. Despite these advantages, important challenges remain, particularly the need for standardised sterilisation procedures, long-term stability, and biocompatibility validation in accordance with ISO 10993-1 [48] guidelines.

Through these attributes, nanomaterials bridge the gap between chemical and physical antibiofilm strategies, enabling durable, safe, and controllable infection-preventive materials for clinical and industrial applications.

### 3.3. Enzymatic Strategies

#### 3.3.1. DNase I Coatings for Extracellular DNA Degradation

Extracellular DNA (eDNA) present within the biofilm matrix plays a crucial structural and adhesive role by binding cells and stabilising the extracellular polymeric network. Therefore, the degradation of eDNA has become a key enzymatic approach for preventing or dispersing biofilms. Deoxyribonuclease I (DNase I) is a well-characterised endonuclease capable of hydrolysing eDNA, thereby destabilising mature biofilms and preventing the adhesion of new bacterial cells [9].

Aktan et al. [9] developed a novel alternating current electrophoretic deposition (AC-EPD) technique for immobilising DNase I on titanium alloy (Ti6Al4V) surfaces. This rapid (10 min) electrochemical process yielded a uniform and dense enzyme layer that retained high catalytic activity. Compared to conventional dip coating, the AC-EPD produced coatings with superior enzyme loading and stability. Release profile studies showed an initial burst of DNase I activity, followed by sustained enzymatic function over several days, making the coating suitable for both early-stage prevention and long-term antibiofilm protection.

In in vitro assays using Staphylococcus epidermidis and *Pseudomonas aeruginosa* models, AC-EPD DNase coatings significantly reduced biofilm biomass compared to uncoated and passively adsorbed DNase-coated surfaces. Crystal violet (CV) staining revealed >80% inhibition of biofilm formation, while confocal laser scanning microscopy (CLSM) confirmed the disaggregation of pre-formed biofilm clusters [9]. Furthermore, the coating retained enzymatic activity after three washing cycles and partial functionality after ethylene oxide (EO) sterilisation [28].

Cytotoxicity testing on fibroblast cultures demonstrated excellent biocompatibility, in accordance with the ISO 10993-5 [49] guidelines. Importantly, the DNase coating activity was non-bactericidal; it dismantled biofilms without directly killing bacteria, thus reducing the selective pressure for resistance development. This “biofilm dispersal” mechanism makes DNase I coatings ideal as a prophylactic barrier for titanium-based implants, orthopaedic screws, and dental devices, where bacterial adhesion precedes colonisation. The study also established that AC-EPD coatings are compatible with complex geometries, suggesting their scalability for industrial applications.

The combined findings of Aktan et al. [9] and Rouchon et al. [28] emphasise that DNase I remains a highly promising component of enzymatic antibiofilm systems, especially when immobilised via electrochemical or covalent methods to ensure long-term functionality and sterilisation stability.

#### 3.3.2. Lysostaphin-Functionalised Silicone Catheters

While DNase I targets the biofilm matrix, lysostaphin acts directly on bacterial cell walls, particularly those of *Staphylococcus aureus* and *S. epidermidis*. Lysostaphin is a zinc metalloendopeptidase produced by *Staphylococcus simulans* that cleaves the pentaglycine bridges unique to staphylococcal peptidoglycan, resulting in rapid cell lysis [5]. Its species selectivity and potent lytic action make it especially useful against *S. aureus* biofilms, which are among the most common causes of catheter- and implant-related infections.

Jayakumar et al. [5] engineered lysostaphin-functionalised silicone catheters through covalent immobilisation using silane–glutaraldehyde coupling. Two coating variants were compared: surface-adsorbed lysostaphin (Lst(A)) and covalently bound lysostaphin (Lst(F)). Both reduced *S. aureus* and *S. epidermidis* colonisation; however, the covalently bound Lst(F) catheters exhibited substantially higher antibiofilm efficacy and durability.

In static in vitro models, both coatings reduced biofilm biomass by >90% within 24 h. Under flow conditions simulating urinary catheterisation, the Lst(F) coating maintained its activity, whereas the adsorbed variant lost its function due to enzyme desorption. Scanning electron microscopy revealed an almost complete absence of biofilm structures on the Lst(F) surfaces, confirming stable surface activity [5].

Crucially, in a murine subcutaneous catheter model, Lst(F)-functionalised catheters achieved sustained protection against staphylococcal infection, exhibiting significantly lower bacterial loads and inflammatory responses than both uncoated and Lst(A)-treated controls. Cytotoxicity and hemocompatibility tests revealed no adverse effects, indicating complete biocompatibility [5].

This study highlights several key design principles for enzymatic coatings: covalent attachment ensures enzyme retention and long-term activity under physiological flow; specificity toward target pathogens reduces collateral effects on commensal flora and combination potential, which means that lysostaphin coatings could be integrated with DNase I or antimicrobial peptides to broaden their activity and enhance their efficacy against mixed-species biofilms.

However, the narrow antibacterial spectrum of lysostaphin limits its use to staphylococcal infections. Future developments could involve hybrid coatings containing multiple enzymes or enzyme–nanomaterial composites to overcome these constraints. Nonetheless, lysostaphin remains one of the few enzymes that consistently demonstrates both in vitro and in vivo antibiofilm efficacy, validating enzymatic functionalisation as a clinically viable strategy for infection-resistant catheters and implants to prevent biofilm formation.

Both DNase I and lysostaphin exemplify how targeted biological catalysis can weaken biofilm defences without the use of antibiotics. The next frontier involves hybrid enzymatic coatings, such as co-immobilisation of DNase I with antimicrobial peptides or silver nanoparticles, to merge biofilm dispersal with bactericidal effects [9,28].

Key priorities include the preservation of enzymatic activity after sterilisation (EO, gamma), integration with flexible polymeric materials beyond titanium and silicone, and standardised ISO 10993-1 [48] biocompatibility and endotoxin testing for regulatory approval.

As these enzymatic platforms evolve, they offer a route toward selective, self-sterilising biomaterials capable of dismantling biofilms with biochemical precision rather than broad-spectrum toxicity.

### 3.4. Physical and Photonic Approaches

#### 3.4.1. Metallic Coatings with Contact-Killing and ROS-Mediated Effects

Physical surface modification using metallic films is one of the most clinically advanced strategies for preventing biofilm formation on surgical and implantable devices. Among these, silver-coated titanium and stainless steel surfaces represent mature and well-validated technologies. Souza et al. [23] deposited thin silver films onto titanium substrates and stainless steel surgical components through physical vapour deposition (PVD) and sputtering, targeting *Staphylococcus aureus*, *Pseudomonas aeruginosa*, and *Escherichia coli* as representative biofilm-forming pathogens.

The antibacterial mechanism combines contact killing and ROS generation. Silver ions (Ag^+^) interact with thiol-containing membrane proteins, disrupting redox balance and protein folding, while surface-bound silver catalyses local ROS formation, promoting oxidative damage to lipids and DNA [23]. In vitro assays demonstrated >90% reduction in both biofilm biomass and viable cell counts after 24–72 h. Importantly, the antibacterial activity persisted through multiple washing, EO sterilisation, and biological fluid exposure cycles without measurable silver loss or mechanical degradation.

The titanium oxide layer beneath the silver film acts as a diffusion barrier that limits ion leaching, thereby improving the long-term safety. Cytocompatibility testing confirmed compliance with ISO 10993-17/-18 [50,51] standards for metallic components, although tight control of Ag^+^ kinetics remains essential to prevent chronic cytotoxicity. The main constraints involve the high cost of deposition and potential colour or gloss alteration; however, the durability, scalability, and clinical validation of Ag-coated Ti surfaces make them an excellent benchmark for emerging hybrid photonic systems.

#### 3.4.2. Photodynamic and Photocatalytic Systems

The convergence of photochemistry and nanomaterials has enabled precise, on-demand biofilm inactivation using light as an external trigger to activate nanomaterials. Cyclometalated Ir (III) complexes (Ir1 and Ir2) exemplified this approach. Upon irradiation at 370 nm, these complexes generate singlet oxygen (^1^O_2_) and secondary ROS that oxidatively damage bacterial membranes, proteins and nucleic acids [40]. After 15 min of illumination, Ir1 and Ir2 achieved a nearly 5 log reduction in planktonic *S. aureus*, including methicillin-resistant *S. aureus* (MRSA)strains, with minimal cytotoxicity toward keratinocytes at low concentrations. In mature, three-day methicillin-susceptible *Staphylococcus aureus* (MSSA) biofilms, biomass decreased by 40–65%, whereas MRSA biofilms showed 20–32% reduction.

The advantage of iridium complexes lies in their light-controlled activation, which confines ROS generation to the illuminated region, thereby minimising systemic toxicity and preventing resistance development. Moreover, these compounds can be immobilised onto metallic or polymeric surfaces, enabling the development of reusable self-disinfecting materials. Nevertheless, limited light penetration through the tissue and potential phototoxicity at higher doses require further in vivo safety studies [40].

Complementary results were reported by Hu et al. [41], who synthesised a rough-surfaced Ag_2_S@H–CeO_2_ nanocomposite integrating photothermal and photodynamic effects. Under near-infrared (NIR) irradiation, Ag_2_S generated localised heat (~45 °C) that increased bacterial membrane permeability, while the CeO_2_ shell, via Ce^3+^/Ce^4+^ redox cycling, exhibited peroxidase-like activity that catalysed ROS formation. This dual action efficiently eradicated bacteria and destroyed biofilms of both MRSA and extended-spectrum β-lactamase (ESBL)-producing *E. coli* [41].

In a murine wound infection model, treatment with Ag_2_S@H–CeO_2_ under NIR illumination significantly decreased the bacterial load and accelerated wound closure without detectable systemic toxicity [41]. The synergistic combination of photothermal heating and catalytic ROS generation, coupled with glutathione depletion that disrupts bacterial redox homeostasis, makes this composite a compelling candidate for photonic-wound-healing platforms. Challenges include the limited tissue penetration of NIR light and the need for long-term biodistribution studies of silver and cerium ions.

#### 3.4.3. Microscale Enzyme–Photocatalyst Robots

An innovative approach merges photochemistry with microscale mobility. Urease-driven TiO_2_/CdS microrobots (U-μrobots) utilise enzymatic propulsion and visible-light activation for autonomous biofilm removal [35]. The urease layer decomposes urea into CO_2_ and NH_3_, creating chemical gradients that propel particles through viscous biofilm matrices. Concurrently, the TiO_2_/CdS core generates ROS under illumination, producing localised oxidative and mechanical disruption of the biofilms.

In vitro experiments against *E. coli* biofilms demonstrated ≈ 90% biomass reduction within two hours of visible light exposure. CLSM and SEM imaging confirmed the fragmentation of the biofilm architecture and cell death throughout the matrix depth. The robots retained motility and catalytic activity over multiple operational cycles at physiological urea levels (0.5–1%).

This enzyme–photocatalyst tandem enables biofilm penetration even in low-oxygen and low-light environments, which are typical of catheter interiors. However, practical application is currently hindered by the complexity and cost of microrobot fabrication, potential CdS phototoxicity, and the lack of in vivo validation [35]. Nevertheless, such active motion systems point toward next-generation self-cleaning biomedical devices capable of autonomous decontamination of tubing, drains, and implant surfaces.

Several unifying themes can be identified across these photonic and metallic systems. A central element of their antimicrobial action is the generation of ROS, which serves as a common mechanism underlying the activity of Ir(III) complexes, Ag/TiO_x_ coatings, Ag_2_S@H–CeO_2_ nanozymes, and TiO_2_-based microrobots. All of these rely on controlled oxidative stress for bacterial inactivation. These approaches also emphasise durability and safety, as hybrid photocatalytic materials demonstrate stability over multiple application cycles while exhibiting minimal ion leaching. However, they still require thorough evaluation in accordance with the ISO 10993-1 [48] photobiological safety standards. An important advantage of these technologies is their on-demand activation, as light-triggered systems allow precise spatial and temporal control, thereby increasing efficacy and reducing the risk of cytotoxicity. Moreover, their design offers a significant integration potential. Photonic platforms can be effectively combined with enzymatic or polymeric anti-adhesive layers to create multimodal adaptive coatings that enhance the overall performance of antimicrobial surfaces.

These approaches demonstrate that physical and photonic activation mechanisms can provide sterilisation without antibiotics while maintaining compatibility with medical-grade polymers and metal implants.

### 3.5. Natural Compounds and Biological Systems

#### 3.5.1. Natural and Plant-Derived Compounds

Natural compounds offer eco-friendly and biocompatible antibiofilm agents. The principal component of eucalyptus oil, 1,8-cineole, disrupts bacterial membranes and interferes with quorum sensing (QS) pathways, leading to 70–90% adhesion reduction across *E. coli*, *S. aureus*, and *P. aeruginosa* without cytotoxicity ≤ 0.5% [2]. Similarly, sulphur- and phenolic-containing Allium extract compounds (e.g., allicin and ajoene) oxidise membrane proteins and suppress QS gene expression (*las, rhl, luxS*), achieving 70–95% biofilm biomass reduction in mixed bacterial and *Candida* biofilms [26]. Polyphenols, such as gallic acid, ferulic acid, and quercetin, synergistically disrupt cell membranes and QS signalling, leading to 80–95% biomass reduction with ≤1% cytotoxicity, although light and oxygen instability limit their shelf life [27].

Biosurfactants, including glycolipids and lipopeptides from *Candida* or *Lactobacillus*, act as amphiphilic, anti-adhesive agents. By lowering the surface tension, they hinder bacterial adhesion and destabilise EPS matrices. *Candida*-derived glycolipid coatings on silicone catheters reduced *E. coli* and *Candida albicans* biofilms by up to 97%, surpassing synthetic surfactant SDS [34]. Their biodegradability, pH stability, and low toxicity make them ideal candidates for passive, non-leaching surface treatments.

#### 3.5.2. Marine Microbial Metabolites

Marine bacteria are unexplored sources of antibiofilm metabolites. *Enterobacter* sp. 84.3, isolated from a marine sponge, produced water-soluble fractions that inhibited and dispersed *S. aureus* and *S. epidermidis* biofilms without affecting their planktonic growth [1]. The aqueous extract exhibited dose-dependent activity (MBEC 16–256 mg/mL) and showed no cytotoxicity to L929 fibroblasts at concentrations up to 500 mg/mL. Confocal microscopy revealed substantial thinning and detachment of staphylococcal biofilms, highlighting this strain as a potential source of non-biocidal, surface-modifying anti-biofilm agents.

#### 3.5.3. Bacteriophage-Based Antibiofilm Systems

Bacteriophage therapy has re-emerged as a promising alternative to antibiotics for controlling biofilm-associated infections, particularly those caused by MDR *Staphylococcus aureus* and *Pseudomonas aeruginosa*. Two complementary approaches, bacteriophage hydrogels and lytic phage isolates, illustrate the progress from basic virology to advanced biomaterial integration.

Zuo et al. [47] developed a nanohydrogel-based bacteriophage delivery system (Phage@Hydrogel) using a chitosan matrix that encapsulates phages within nanocapsules. The hydrogel is designed to respond to local microenvironmental cues characteristic of biofilms, such as acidic pH and enzymatic activity, which trigger controlled phage release. This design maintains phage viability for at least seven days under physiological conditions while protecting them from enzymatic degradation and desiccation [47].

Upon release, bacteriophages infect bacterial cells, propagate through lytic replication, and progressively dismantle the biofilm structure. In in vitro assays, Phage@Hydrogel achieved a 4–5 log reduction in viable cell counts and biomass of *P. aeruginosa* and *S. aureus* biofilms within 24–48 h. Confocal microscopy has confirmed deep biofilm penetration and structural disruption [47]. In in vivo wound infection models, hydrogel-treated groups exhibited accelerated wound closure and reduced bacterial load compared to free-phage and control treatments.

The system offers several advantages: localised action, reduced systemic exposure, protection of phages during storage and sterilisation, and compatibility with dressings and implants. However, their limitations include a narrow phage host range, potential immunogenicity, and production challenges related to long-term stability. For clinical translation, phage-based hydrogels must undergo ISO 10993-5 [49] compliant cytotoxicity, sensitisation, and residual safety testing, as well as MDR 2017/745 conformity.

Complementing this platform, Suchithra et al. [46] isolated and characterised RuSa1, a novel lytic *Staphylococcus aureus* bacteriophage of the Kayvirus genus. Isolated from a wastewater source, RuSa1 displayed an icosahedral head (70–80 nm) and contractile tail (~160 nm). Its short latent period (10 min) and high burst size (~50 PFU/cell) indicate a strong lytic potential. RuSa1 lysed 18 of 20 clinical MRSA isolates, with no activity against *P. aeruginosa*, *E. coli*, or *Enterococcus faecalis*, confirming its narrow host specificity [46].

In biofilm assays, RuSa1 inhibited early *S. aureus* biofilm formation by >88% and degraded mature biofilms by up to 76%. Fluorescence microscopy confirmed the extensive loss of viable cells. Genome analysis revealed a 140 kb dsDNA genome lacking lysogenic, virulence, and antibiotic resistance genes, confirming its therapeutic safety. The phage retained infectivity for 60 days at 25 °C and remained stable between pH 5 and 9 and 4–37 °C.

Together, Phage@Hydrogel and RuSa1 exemplify the growing integration of biological precision with materials engineering, offering targeted, eco-friendly, and self-amplifying antibiofilm solutions suitable for wound care and implant protection.

#### 3.5.4. Biosurfactants and Molecules of Microbial Origin

Naturally produced biosurfactants, such as rhamnolipids and lipopeptides, provide amphiphilic structures that reduce surface tension, hinder bacterial adhesion, and destabilise the EPS matrix. Ceresa et al. [20] demonstrated that microbial biosurfactants effectively inhibit the biofilm formation of *S. aureus*, *S. epidermidis*, *P. aeruginosa*, and *Candida albicans* on titanium and polymeric surfaces.

Their dual mechanism involves physicochemical surface modification and biochemical disruption of the biofilm integrity. Biosurfactants reduced surface hydrophobicity, interfered with protein–polysaccharide interactions, and caused 60–90% biomass reduction, depending on the concentration and strain. Confocal microscopy confirmed the thinning and fragmentation of biofilms, while cytotoxicity tests on fibroblasts and keratinocytes showed no toxicity at ≤0.05 mg/mL [20].

The advantages of these compounds include their natural origin, biodegradability, and minimal resistance development. However, batch variability and storage instability necessitate production standardisation. The compatibility of biosurfactants with polymer coatings and hydrogels suggests their potential integration into regenerative or cleaning systems, where they can function as both surfactants and biocidal modulators.

### 3.6. Hybrid Bio-Material and Bio-Physical Systems

#### 3.6.1. Hybrid Enzymatic and Photocatalytic Systems

Hybrid biological–physical systems combine enzyme selectivity with external activation stimuli such as light or ultrasound. One of the most sophisticated examples is the urease–photocatalyst microrobot (U-μrobot) developed by Villa et al. [35], which was described previously in the photonic section. Its hybrid mechanism, which involves biochemical propulsion via urease and photocatalytic ROS generation, illustrates how biological components can provide autonomous navigation, while inorganic elements deliver controlled disinfection [35].

Another notable example is sonodynamic materials with adaptive redox control. Huang et al. [45] designed a BiVO_4_/multi-walled carbon fullerene (MCF) heterostructure capable of bidirectional ROS modulation under ultrasound stimulation. During sonodynamic activation, the BiVO_4_ component generated bactericidal ROS, eradicating MRSA biofilms with 99.9% efficiency in vitro. In contrast, under resting conditions (no ultrasound), the same material scavenges excess endogenous ROS and protects the tissue from oxidative stress [45].

In diabetic mouse wound models, BiVO_4_/MCF combined with ultrasound therapy downregulated inflammatory cytokines (IL-6 and TNF-α to ~2% baseline) and upregulated angiogenic markers vascular endothelial growth factor (VEGF) and cluster of differentiation 31 (CD31), promoting neovascularisation and accelerating healing [45]. The ability to alternate between oxidative and antioxidant functions marks a paradigm shift toward intelligent, self-regulating, antibiofilm biomaterials.

#### 3.6.2. Nanobubble and Fluidic Antibiofilm Systems

An unconventional but increasingly relevant physical–biological hybrid involves the use of micro- and nanobubbles (NBs). These gaseous nanostructures, typically <1 μm in diameter, exhibit unique physicochemical properties, such as high surface charge (zeta potential), long lifetime, and spontaneous ROS generation during cavitation events.

Singh et al. [44] reviewed multiple applications of nanobubbles in water treatment, irrigation systems, and bioreactors, highlighting their antibiofilm and antifouling potential. In hydrodynamic systems, nanobubbles generated via ultrasonic cavitation or Venturi mixing enhance oxygen transfer and induce localised ROS bursts that can disrupt bacterial films [44].

In membrane-based processes, NaHCO_3_-modified thin-film polyamide membranes with integrated NBs showed reduced fouling and improved solute rejection, albeit with decreased flux. Similarly, electro-ceramic membranes incorporating nanostructured carbon or zeolite coatings demonstrated self-cleaning functionality through periodic electrolysis, which generated hydrogen microbubbles, detaching sodium alginate and yeast foulants, and restoring 90–95% of the original flux [44].

These technologies underscore the expanding frontier of bubble-assisted antibiofilm control, where controlled cavitation replaces chemical cleaning, reducing both the cost and environmental burden. Opportunities include integration into dialysis circuits, medical tubing, and water sterilisation systems to maintain sterility without chemical agents.

## 4. Conclusions

The increasing challenge of antimicrobial resistance, particularly in biofilm-related infections, necessitates a shift from conventional antibiotics to integrative and multifunctional strategies. This review highlights a wide array of material-based antibiofilm innovations in the chemical, enzymatic, biological, and physical domains. The core message is clear: the future of infection control lies in intelligent, localised, and adaptive systems that minimise toxicity while maintaining their long-term effectiveness.

Chemical systems such as silver-based coatings, nitric oxide-releasing peptides, and biosurfactants exhibit strong bactericidal and anti-adhesive properties. Notably, biosurfactants, despite their physicochemical activity, serve as a hybrid biochemical interface owing to their microbial origin. Enzymatic systems, including DNase I and lysostaphin, selectively degrade biofilm matrices without promoting resistance, offering a biologically specific mode of action. Emerging biological approaches, such as biosurfactants, bacteriophage hydrogels, and plant-derived metabolites, emphasise sustainability and compatibility, presenting promising avenues for developing long-term antimicrobial strategies.

Photocatalytic and nanomaterial-based physical systems further enhance the set of available approaches through light-triggered, non-leaching, and on-demand sterility. Examples include Ag/TiO_x_ catalysts, Ir(III) complexes, and microrobots incorporating enzyme–photocatalyst hybrids, which demonstrate efficient biofilm removal with reusable and targeted functionality.

Three notable innovations show strong application potential in the medical sector: the nitric oxide-releasing peptide FOTyr-AMP, nanozyme-based CPP system, and urease-powered microrobots (U µrobots). These systems have achieved significant in vivo efficacy in models of device-related infections, surpassing the efficacy of traditional antibiotics in certain applications. Their ability to respond dynamically to physiological triggers, such as light and urea, highlights their potential for next-generation adaptive medical device coatings.

However, clinical implementation requires further validation under ISO 10993-1 [48] biocompatibility standards, robust sterilisation protocols, and long-term performance testing under physiologically relevant conditions. Regulatory integration, particularly with frameworks such as MDR 2017/745, is essential for their transition into practical use.

In summary, the evidence presented illustrates a shift from conventional antibiotic interventions toward forward-looking, smart material-driven approaches for antibiofilm management. These emerging systems, which integrate advances in chemistry, biology, and nanotechnology, have the potential to revolutionise biofilm management in modern medicine by enabling self-sterilising, biocompatible, and dynamically responsive surfaces for biofilm control.

## Figures and Tables

**Table 1:** Inclusion and exclusion criteria were applied in the study selection process.

Inclusion	Exclusion
Describes the type of innovation used	Not published in English
Explains the mechanism of action	Full text not available
Specifies the application context (e.g., clinical or experimental, including medical devices)	Focuses on biofilms outside the medical sector (e.g., food industry)
Reports effectiveness outcomes (quantitative or qualitative)	Does not describe an innovative approach

**Table 2:** Comparative matrix of biofilm prevention and eradication strategies in medical settings.

Author, Year	Type of Innovation	Mechanism of Action (Brief)	Device/Context	Type/Efficacy Evaluation	Advantages	Limitations
Selem et al., 2022 [4]	Nanomaterials (AgNPs)	Anti-adhesive and bactericidal via ROS generation	Urinary catheters	In vitro; marked reduction in *E. coli* biofilm	Simple and effective material; broad antimicrobial spectrum	Potential cytotoxicity and uncontrolled Ag release
Aktan et al., 2022 [9]	Enzyme-coated surface (DNase I via AC-EPD)	Biofilm matrix degradation	Titanium discs (implant surface model)	In vitro; reduced biomass of *S. aureus* and *E. coli*	Targeted action; feasible electrodeposition method for implant coating	Limited enzyme stability; high production cost
Jayakumar et al., 2024 [5]	Enzymatic (lysozyme-like lysostaphin immobilised on silicone)	Cell wall lysis within biofilm	Silicone catheters	In vitro; strong activity against *S. aureus* biofilm	High specificity toward *Staphylococcus* spp.	Narrow antimicrobial spectrum
Ibanez-Cervantes et al., 2023 [10]	Physical technology (ozone, O_3_)	Oxidative biofilm disruption and degradation	Respirators/ventilator pathways	In vitro; reduced *P. aeruginosa* biofilm	Antibiotic-free approach; rapid antimicrobial action	Implementation and safety challenges in respiratory systems
Ciandrini et al., 2020 [11]	Antimicrobial peptides (AMPs)	Membrane disruption and anti-adhesive activity	CVC surfaces/polystyrene substrates	In vitro; inhibition of *S. aureus* biofilm formation	Novel class of bioactive molecules	Limited stability; high production cost; potential hemolytic effects
Vento et al., 2024 [12]	Nano-porphyrin (photodynamic therapy, PDT)	ROS-mediated bactericidal and biofilm-eradication effect (light-activated)	General medical devices	In vitro; eradication of *S. aureus* biofilm	Synergistic nano–light mechanism; high antimicrobial efficacy	Requires external light source; limited clinical translation so far
Nunes et al., 2021 [1]	Post-/probiotic approach (cell-free supernatant, CFS)	Competitive inhibition and interference with bacterial adhesion	Implantable/orthopaedic devices	In vitro; reduced surface colonisation	Natural origin; low cytotoxicity	Variable composition; unclear mechanism of action
Bletsa et al., 2023 [3]	Photocatalytic coatings (Ag/TiO_x_)	ROS generation and anti-adhesive surface activity	Medical device surfaces	In vitro; reduction in both Gram-positive and Gram-negative bacteria	Durable and long-term antimicrobial activity	Potential cytotoxicity related to metal release
Peter at al., 2023 [13]	Hydrophilic coatings with AgNPs	Anti-adhesive and bactericidal effects	Gloves, catheters, stethoscopes	In vitro; reduced bacterial adhesion and growth	Simple application to various medical devices	Limited coating durability under real-use conditions
Salazar-Sesatty et al., 2024 [14]	Magnetic nanocarriers with curcumin (nano-CS)	Targeted drug delivery and bactericidal activity	Medical implants	In vitro; strong biofilm reduction	Smart carrier system with natural compound; biocompatible	Early development stage; scalability and dosage control issues
Klubthawee et al., 2023 [15]	Hybrid antimicrobial peptide (CM-10K14K)	Bactericidal and anti-adhesive surface activity	Foley catheters	In vitro; strong reduction in *E. coli* biofilm	Synthetic, biofilm-targeted peptide design	Limited stability and retention of activity in vivo
Yu et al., 2021 [16]	PDA/uhPDMA superhydrophilic coating	Anti-adhesive effect; reduced bacterial colonisation	Catheters	In vitro; minimal or no bacterial adhesion	Biocompatible and durable surface coating	Lack of clinical validation data
Mayorga-Martinez et al., 2021 [17]	Micromotors (“Aqua Sperm”)	Mechanical scraping and biofilm disruption	Tubing/flow-line systems	In vitro; effective biofilm removal	Highly innovative physical removal strategy	Early proof-of-concept; complex implementation
Agarwal et al., 2021 [18]	SLIPS surface with controlled triclosan release	Anti-fouling and localised antimicrobial release	Tubing and medical device surfaces	In vitro; high resistance to biofouling	Advanced surface engineering; long-lasting effect	Regulatory restrictions associated with triclosan use
Fei et al., 2020 [19]	Peptide FOTyr-AMP (NO donor)	Nitric oxide release; biofilm disruption	Medical devices/implants	In vitro; significant biomass reduction	Multifunctional NO activity; anti-quorum sensing effect	Dose control and peptide stability challenges
Ceresa et al., 2021 [20]	Biosurfactants (lipopeptides/rhamnolipids)	Anti-adhesive effect; biofilm matrix destabilisation	Implant-mimicking discs	In vitro; reduced multispecies adhesion	Natural, metabolically derived agents	Variable composition; limited scalability
Lu et al., 2023 [21]	Anti-biofilm peptide (e.g., RK22)	Disruption of biofilm formation and planktonic cell activity	General medical device applications	In vitro; inhibition of biofilm development	Specifically targeted antibiofilm peptide	Limited peptide stability and production efficiency
Ansari et al., 2021 [22]	Ni–Cu–Zn nanoferrites (NSFs)	Contact-based bactericidal and anti-adhesive activity	General medical device applications	In vitro; decreased CFU count and biofilm biomass	Durable material with magnetic properties	Potential cytotoxicity due to metal ion release
Souza et al., 2024 [23]	Silver-coated titanium surfaces	Bactericidal and anti-adhesive activity (Ag-based)	Implants and surgical instruments	In vitro; significant biofilm reduction	Mature and well-established coating technology	Risk of silver release; potential biocompatibility concerns
Imani et al., 2022 [24]	Bio-nanocomposite (alginate/kaolin/Ag)	Barrier formation and bactericidal activity (Ag-based)	Dental materials	In vitro; reduced colonisation by *S. mutans*	Synergistic effect of matrix and silver components	Trade-off between durability and cytotoxicity
Kriechbaumer et al., 2020 [25]	Physical technology (Er:YAG laser)	Mechanical and thermal removal of biofilm biomass	orthopaedic plates and pins	In vitro/proof-of-concept; clear surface decontamination	Chemical-free method; rapid action	Requires specialised equipment; safety control for adjacent tissues
Vazquez et al., 2022 [2]	Natural small molecule (e.g., 1,8-cineole)	Membrane disruption and anti-adhesive activity; inhibition of biofilm-associated growth	Urinary catheters/polymeric surfaces	In vitro; decreased CFU and biofilm biomass	Inexpensive and widely available compound	Variable efficacy; lack of in vivo data
Galdiero et al., 2020 [26]	Plant-derived natural metabolites (*Allium* extracts)	Anti-adhesive effect; possible quorum-sensing interference	General medical device context	In vitro; reduced surface colonisation	Natural origin; low cytotoxicity	Variability in composition; difficult standardisation
Martínez Chamás et al., 2023 [27]	Plant-derived phenolic compounds	Inhibition of biofilm formation; antioxidant activity	General medical device surfaces	In vitro; biofilm reduction in both Gram-positive and Gram-negative bacteria	High chemical diversity; low production cost	Limited stability; lack of translational studies
Rouchon et al., 2022 [28]	Enzymes (lysozyme: hen egg white/recombinant human)	Peptidoglycan degradation; biofilm weakening	Catheters/surgical implants	In vitro; reduced adhesion and biofilm biomass	Biological selectivity; potential for surface immobilisation	Enzyme stability and production cost
Pradhan et al., 2024 [29]	Nanomaterial-based antibiofilm formulation	Anti-adhesive and contact bactericidal activity	Medical catheters	In vitro; reduced microbial colonisation	Easy coating process; compatible with polymeric substrates	Limited coating durability under practical use conditions
Tarabal et al., 2024 [30]	Cationic polymer (DMPEI) coating on PVC	Anti-fouling and anti-adhesive activity via cationic interaction	PVC catheters	In vitro; significant reduction in bacterial adhesion	Simple application to polymeric devices	Potential cytotoxicity of cationic agents; regulatory limitations
Firdausy et al., 2024 [31]	Photocatalytic coatings (Ag@TiO_2_/Ag@N–TiO_2_)	ROS generation under light activation combined with Ag-based bactericidal effect	Urinary catheters	In vitro; strong biofilm reduction	Long-term antimicrobial activity; high coating stability	Control of Ag release; dependence on UV/visible light activation
Riahi et al., 2024 [32]	Modified chitosan biopolymer	Anti-adhesive effect through polymeric barrier formation	Surgical instruments/catheters	In vitro; reduced microbial colonisation	Biocompatible and intrinsically antibacterial material	Limited long-term stability and mechanical durability
Noach et al., 2023 [33]	Zinc salt (ZnCl_2_) incorporated into silicone	Zn^2+^ ion release inhibiting bacterial growth and adhesion	Silicone nasal splints	In vitro; reduced biofilm formation	Simple, low-cost component enabling local ion delivery	Potential irritation; control of dosage and leaching required
Bastos et al., 2024 [34]	Biosurfactant (glycolipid from *Candida* sp.)	Anti-adhesive activity and biofilm matrix destabilisation	Siliconised latex catheters	In vitro; decreased bacterial adhesion and biofilm biomass	Natural origin; simple coating process	Variability in composition; challenges in standardisation
Liang et al., 2020 [6]	Nanozyme/enzyme-based catalytic system	ROS generation and biofilm matrix degradation	Medical implants	In vitro; reduction in multispecies biofilm	High catalytic activity; suitable for surface immobilisation	Control of catalytic activity and biosafety required
Villa et al., 2022 [35]	Micromotors (U-μrobot)	Mechanical scraping and biofilm dispersion	Urinary catheters	In vitro; effective biofilm removal	Highly innovative; directionally controllable	Complex implementation and regulatory challenges
Verma et al., 2023 [36]	Nano-SiO_2_ functionalised with resveratrol and glutathione (GSH)	Anti-adhesive and antioxidant surface modification	Silicone Foley catheters	In vitro; reduced bacterial adhesion and biofilm biomass	Smart surface modification combining physical and biochemical effects	Stability of surface loading and control of release kinetics
Poyil et al., 2022 [37]	Natural extract (*Illicium verum*)	Membrane disruption and quorum-sensing interference; anti-adhesive effect	Indwelling urethral catheters	In vitro; decreased CFU count and biofilm biomass	Low-cost and widely available natural compound	Variable potency; lack of in vivo validation
Xiao et al., 2024 [38]	Novel small molecule (maleimido-diselenide YH7)	ROS modulation and antioxidant-based antibiofilm activity	Medical implants	In vitro; strong inhibition of biofilm formation	Represents a new chemical class with dual redox–antibiofilm action	Early proof-of-concept; toxicological profile yet to be established
Aktan et al., 2024 [39]	Modified biopolymer (MA–chitosan)	Anti-adhesive barrier with cationic surface interactions	Ti6Al4V dental implants	In vitro; reduced microbial colonisation	Biocompatible material; simple coating process	Limited long-term stability under oral environmental conditions
Fallon et al., 2025 [40]	Photoactive iridium complexes	PDT/PTT-like ROS generation causing biofilm damage	Catheters/surgical instruments	In vitro; eradication of MRSA biofilm	Photo-functional compounds enabling precise activation	Requires external light source; potential phototoxicity
Hu et al., 2024 [41]	Nanocomposite (Ag_2_S@H–CeO_2_)	ROS generation via CeO_2_ redox cycling combined with Ag-based bactericidal effect	Wound treatment applications	In vitro; marked biofilm reduction	Synergistic interaction of components; high material stability	Control of ion/metal release required
Jang et al., 2020 [42]	Graphene–AgNP hybrid coating	Membrane damage and anti-adhesive surface effect	Catheters/implants	In vitro; decreased bacterial adhesion and CFU count	Strong surface-driven antibacterial activity	Potential cytotoxicity and metal ion release
Padmavathi et al., 2025 [43]	PDMS functionalised with capsaicin	Anti-adhesive effect through surface energy modification	PDMS-based biomedical implants/devices	In vitro; reduced microbial colonisation	Simple and cost-effective polymer additive	Long-term stability of surface loading and biocompatibility over time
Singh et al., 2025 [44]	Electro–self-cleaning membranes	Electrochemically generated ROS enabling surface decontamination	Sterilisation systems/medical fluid pathways	In vitro/proof-of-concept; effective biofilm removal	Chemical-free and on-demand cleaning mechanism	Power supply requirements and electrical safety considerations
Huang et al., 2025 [45]	Ultrasonically activated BiVO_4_/MCF composite	Ultrasound-triggered ROS generation leading to biofilm destruction	Coatings/inserts for medical devices	In vitro; strong antibiofilm effect	Precisely controllable and remotely activated system	Requires ultrasound equipment; heat generation control needed
Suchithra et al., 2025 [46]	Bacteriophage RuSa1	Targeted bacterial lysis within biofilm	General medical device context	In vitro; decreased CFU and biofilm biomass	High specificity toward target bacteria	Narrow host range; potential for phage resistance
Zuo et al., 2022 [47]	Encapsulated bacteriophages	Enhanced stability and improved biofilm penetration	General medical device context	In vitro; high antibiofilm activity	Improved delivery and prolonged phage viability	Challenges in standardisation and large-scale production; potential immunogenicity

## Data Availability

No new data were created or analyzed in this study.

## Abbreviations

The following abbreviations are used in this manuscript:

AgNPs	Silver nanoparticles
MDR	Multidrug-resistant
ROS	Reactive oxygen species
MIC	Minimum inhibitory concentration
CV	Crystal violet
SEM	Scanning electron microscopy
TEM	Transmission electron microscopy
EO	Ethylene oxide
QS	Quorum sensing
EPS	Extracellular polymeric substances
AMP	Antimicrobial peptide
PVC	Polyvinyl chloride
CLSM	Confocal laser scanning microscopy
MBEC	Minimum biofilm eradication concentration
eDNA	Extracellular DNA
AC-EPD	Alternating current electrophoretic deposition
NIR	Near-infrared (light)
MRSA	Methicillin-resistant *Staphylococcus aureus*
IL-6	Interleukin-6
TNF-α	Tumour necrosis factor alpha
MDR 2017/745	EU Medical Device Regulation
